# Structural deformation upon protein-protein interaction: A structural alphabet approach

**DOI:** 10.1186/1472-6807-8-12

**Published:** 2008-02-28

**Authors:** Juliette Martin, Leslie Regad, Hélène Lecornet, Anne-Claude Camproux

**Affiliations:** 1 Equipe de Bioinformatique Génomique et Moléculaire, INSERM UMRS726/Université Denis Diderot Paris 7, F-75005 Paris, France; 2 Unité Mathématiques Informatique et Génome UR1077, INRA, F-78350 Jouy-en-Josas, France

## Abstract

**Background:**

In a number of protein-protein complexes, the 3D structures of bound and unbound partners significantly differ, supporting the induced fit hypothesis for protein-protein binding.

**Results:**

In this study, we explore the induced fit modifications on a set of 124 proteins available in both bound and unbound forms, in terms of local structure. The local structure is described thanks to a structural alphabet of 27 structural letters that allows a detailed description of the backbone. Using a control set to distinguish induced fit from experimental error and natural protein flexibility, we show that the fraction of structural letters modified upon binding is significantly greater than in the control set (36% versus 28%). This proportion is even greater in the interface regions (41%). Interface regions preferentially involve coils. Our analysis further reveals that some structural letters in coil are not favored in the interface. We show that certain structural letters in coil are particularly subject to modifications at the interface, and that the severity of structural change also varies. These information are used to derive a structural letter substitution matrix that summarizes the local structural changes observed in our data set. We also illustrate the usefulness of our approach to identify common binding motifs in unrelated proteins.

**Conclusion:**

Our study provides qualitative information about induced fit. These results could be of help for flexible docking.

## Background

Most of biochemical reactions inherent to the life of a cell are mediated by protein-protein interactions, e. g. the recognition of a substrate by an enzyme, or an antigen by an antibody. Protein-protein interaction is influenced by several factors like the size and shape of the interface, shape complementarity between interacting proteins or hydrophobicity [1,2]. Interfaces between interacting proteins have been extensively studied for decades now [3,4]. It has been shown that they have distinct features when compared to non-specific interfaces observed in protein crystals [5-9], or when compared to the rest of the protein surface [10-16]. Different models have been proposed for the protein binding process. The first was the 'lock and key' model, stating that interacting proteins bind to each other thanks to shape complementarity, without structural modification. Another model has then been suggested: the induced fit, in which the protein structure is modified upon binding [17]. Finally, it is thought that unbound protein exist as an ensemble of conformations, some of them being more favorable for the interaction [18], this is the pre-existing equilibrium model. As the number of experimental 3D protein structures increases, some evidences of induced-fit and pre-existing equilibrium are now available and are described in [19].

The prediction of protein-protein interactions is a current challenge. Some bioinformatic methods have been developed in order to predict whether or not two proteins interact [20-25]. When it is known that two proteins interact, docking methods are employed to predict the 3D structure of the resulting complex, given the structures of interacting partners [26,27]. The performance of docking methods are monitored by Critical Assessment of Predicted Interactions (CAPRI), a blind prediction experiment where structural biologists provide unpublished experimental complex structures as targets for docking programs [28]. Induced fit introduces a supplementary difficulty to the challenging task of docking. Slight modifications involve the rearrangement of side chains that change their conformations to accommodate the interaction with the interacting protein. Stronger modifications can also alter the backbone conformation. Flexible protein-protein docking methods are thus developed in order to account for these conformational changes (see for example [29] for a review of flexible docking methods).

The extend of induced fit modification in protein-protein complexes has been previously studied. A study made by Betts and Sternberg in 1999 revealed that, in a dataset of 39 protein-protein complexes, a half exhibited substantial movements, when compared to pairs of similar proteins solved by different groups [30]. It has been later shown that the structural changes upon protein-protein binding correlate well with the theoretical displacements derived from normal mode analysis [31]. Recently, this was further explored on antibodies that bind different antigens [32]. The case of enzymes has also been addressed: the conformational modification induced by the binding appears to be small in most enzymes (less than 1 Å rmsd), but residues belonging to the binding site exhibit larger backbone motions [33]. Recently, Daily and Gray have used control sets to distinguish between enzyme induced fit modifications and experimental error or intrinsic flexibility of proteins [34]. They found that about 20% of the residues exhibit substantial conformational changes and noted a significant bias toward weakly constrained regions, e. g., loops.

In this study, we propose an investigation of structural changes in protein complexes, from a local point-of-view, *via *a structural alphabet developed in our lab. We consider a set of protein-protein complexes for which the crystallographic structures of both the complex and free partners are available, and quantify the structural changes in terms of structural letter modifications. We also use a control set of 14 protein pairs for which the structures has been independently determined by a different team, as in [34]. The correlation between global change and the number of local change is investigated. We then study the preference for particular letters in the interface regions, and analyze the structural letter substitutions that occur at the interfaces. We also use this new approach to detect common binding motifs in unrelated proteins.

## Results and Discussion

Proteins from 62 complexes are represented as sequences of structural letters using our structural alphabet called HMM-SA [35-37]. We then analyze the differences between bound and unbound structural letter sequences. For clarity, we briefly present here the structural alphabet HMM-SA; more details can be found in [35-37].

HMM-SA, is a library of 27 structural prototypes of four residues, called structural letters, established using hidden Markov model formalism. Thanks to HMM-SA, the 3D structure of a protein backbone is simplified into a sequence of structural prototypes. The simplification relies on C*α *positions only: each four-residue fragment of the protein structure is described by four inter-C*α *distances. The resulting distances are the input of a hidden Markov model, and the structure is translated as a sequence of structural letters. This encoding is made using the Viterbi algorithm [38] and takes into account both the similarity of the fragments with the 27 structural letters and the preferred transitions between structural letters. A protein structure of *N *residues is then encoded as a sequence of *N *- 3 structural letters. The model has been trained on 1429 X-ray structures of globular proteins, presenting less than 30% sequence identity with a resolution better than 2.5 Å and longer than 30 residues. These structures were taken from the PDB, irrespective of their quaternary structures. They represent a total of 332,493 four-residue fragments.

**Figure 1:**
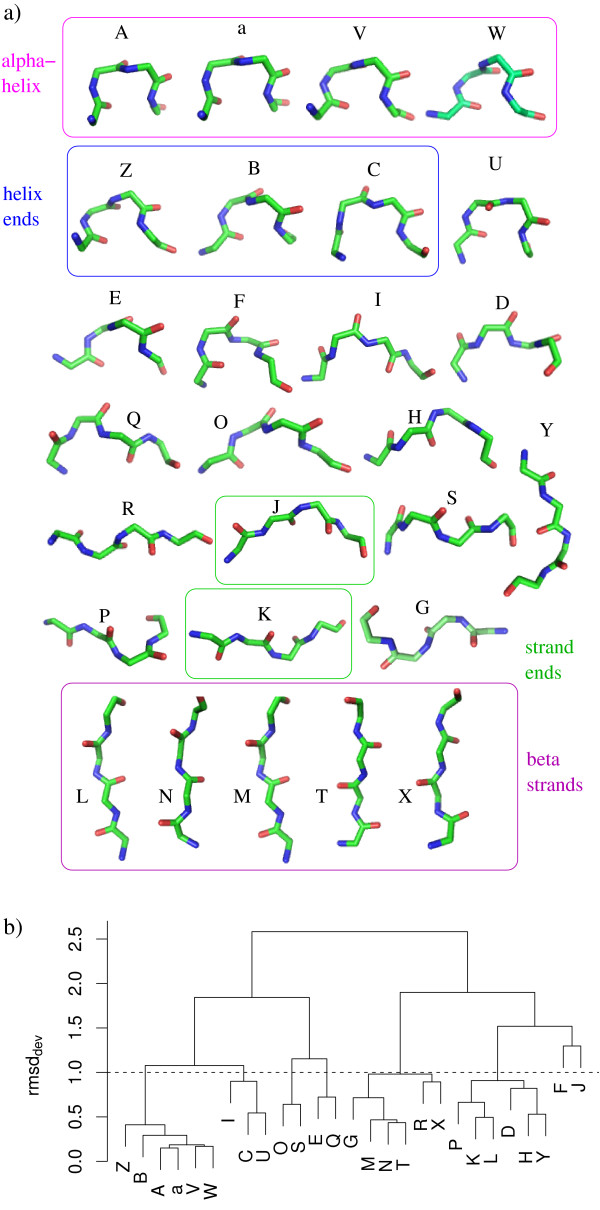
**Presentation of HMM-SA**. a) 3D structure of the 27 structural letters. Images are generated using pymol [54]. b) hierarchical clustering of the 27 structural letters using the rmsd_*dev*_.

The 27 structural letters, named [A-Z, a], are shown on Figure 1a. It has been shown previously [37], that four structural letters, [a,A,V,W] specifically describe *α*-helices, and five structural letters, [L,M,N,T,X], specifically describe *β*-strands. Letters [a] and [A] are the most regular, [a] being slightly shorter. It has been shown that linear helices are encoded by runs of [A], and curved helices are encoded by runs of [a] [39]. The 18 remaining structural letters describe loops. Letters [Z,B,C] form helix ends and letters [J,K] form strand ends. This alphabet allows a very precise decomposition of 3D structures. Some structural letters are structurally close, while others are more distant. This is quantified using the root-mean-square deviation between two structural letters (rmsd_*dev*_). The rmsd_*dev *_has been computed from 500 fragment pairs randomly chosen in the two structural letters [36]. It has been shown, that the rmsd_*dev *_between two structural letters is always greater than the intrinsic variability of each structural letters, measured in the same way and called rmsd_*intra *_[36]. Figure 1b reports the hierarchical clustering of the 27 structural letters according to the rmsd_*dev*_. Using a cut-off of 1 Å, the 27 structural letters are grouped into 8 groups: [Z,B,A,a,V,W], [I,C,U], [O,S], [E,Q], [G,M,N,T,R,X], [P,K,L,D,H,Y], [F], and [J].

### Number of local changes

A set 14 protein pairs solved by different groups is used as a *control set *to assess the local structural changes observed in a data set of 68 protein-protein complexes, denoted as the *complex set*. In the *control set*, 1,128 structural letters are modified out of 4,072(28%). The total number of structural letter pairs considered in the *complex set *is 32,356. Overall, 20,679 structural letters are unchanged (64%), and 11,677 are changed (36%). This proportion is significantly greater than the proportion in the *control set*, as assessed by a Chi-square test (p-value < 2.10^-^16).

If we consider the 8 groups of structural letters with a 1 Å rmsd_*dev *_threshold (described above and shown in Figure 1) by ignoring structural letter changes within the same group, we obtain the following results: 5% of the *control set *is modified (216 modifications out of 4,072) and 11% of the *complex set *is modified (3,666 modifications out of 32,356). These proportions are significantly different as assessed by a Chi-square test (p-value < 2.10^-^16).

The global compositions of bound and unbound chains, in terms of structural letters, are similar (data not shown), except for helical letters [A] and [a]: bound conformations have more [a] and less [A] than unbound conformations.

### Number of changes in different classes

There are 3 classes of complexes in the *complex set*: enzyme-substrate, antibody-antigen, and others. Table 1 summarizes the number of changes in the different types of complexes.

**Table 1:** Percentage of modified structural letters in the complex set

	Type of complex											
	Enzyme-substrate			Antibody-antigen			Others			All types		
rmsd range^*a*^	${\text{N}}_{C}^{b}$	${\text{N}}_{T}^{c}$	%^*d*^	*N*_*C*_	*N*_*T*_	%	*N*_*C*_	*N*_*T*_	%	*N*_*C*_	*N*_*T*_	%

less than 1 Å	29	5145	29%	17	2937	22%	45	8358	34%	91	16440	30%
1 to 2 Å	18	2786	36%	11	2539	38%	28	6245	42%	57	11570	40%
2 to 3 Å	4	509	42%	0	-	-	11	2485	47%	15	2994	46%
more than 3 Å	0	-	-	2	425	29%	6	927	61%	8	1352	51%

All rmsd	51	8440	32%	30	5901	29%	90	18015	40%	171	32356	36%

Control set	19	4072	28%									

^*a*^: C*α *root mean square deviation between bound and unbound conformations, ^*b*^: number of protein chains involved, ^*c*^: total number of structural letters involved, ^*d*^: percentage of structural letters that differ between bound and unbound conformation.

Antibody-antigen complexes undergo 29% of structural letter modifications, a number similar to that obtained on the *control set*. Thus, on the limited number of structures available (10 antibody-antigen complexes), this class of proteins shows only moderate modifications upon protein-protein binding. The 'other' class experiences the highest percentage of structural letter changes (40%). This class encompasses different kind of complexes (transport proteins, signaling proteins, viral capsid). The enzyme class has an intermediate behavior with 32% of modifications. In their study, Daily et al found that 20% of the residues in enzymes are significantly modified upon binding [34]. Here, we find 32% of change in the structural letter sequences. As we show later, a part of these changes replace a structural letter by a similar one. Table 1 also reports the percentages of modified structural letters according to the root mean square deviation of the C*α *(C*α *rmsd). For all types of complexes, the global tendency is a correlation between the percentage of modified structural letters and the C*α *rmsd, the exception being the antibody-antigen in which a low percentage of modified letter (29%) is obtained for high rmsd (more than 3Å) for 2 chains.

### Local change versus global change

Some structures undergo minor global modifications, and other structures are significantly modified, as assessed by the C*α *rmsd ranging from 0.2 to 14.0 Å. For comparison, the C*α *rmsd on the *control set *ranges from 0.20 to 0.38 Å, with a mean value equal to 0.30 Å (same rmsd is obtained for allosteric and non-allosteric protein pairs). The percentage of modified structural letters for different rmsd ranges is indicated in Table 1. As expected, the percentage of modified structural letters is higher on the structures with high rmsd.

**Figure 2:**
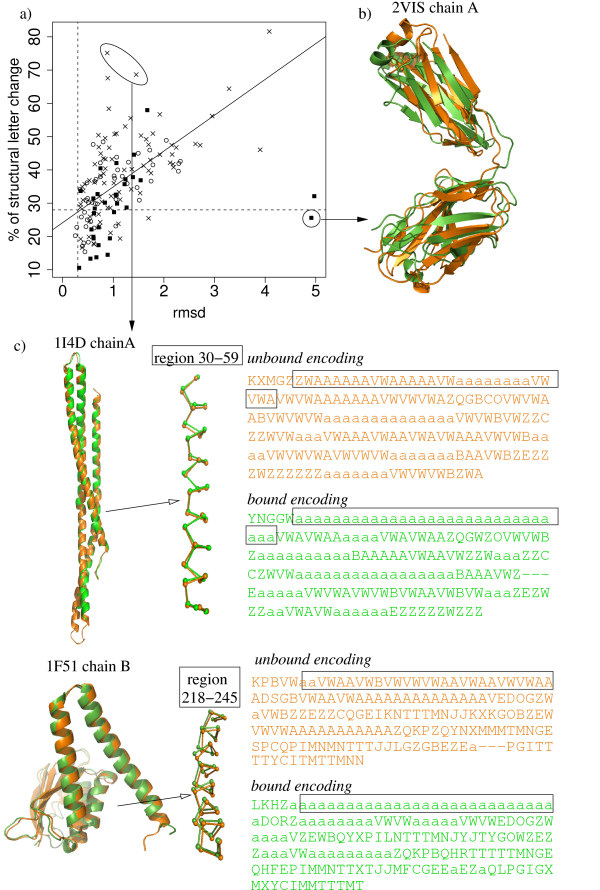
**Correspondence between global and local changes in bound/unbound chains**. a) C*α *rmsd (x axis) versus percentage of modified structural letters (y axis). Open circles: enzyme-substrate, plain squares: antigen-antibody, crosses: other. One chain of complex 1H1V, with a rmsd equal to 14 Å and 55% of modified structural letters is outside the range of this plot. The regression line shown on the plot is obtained by excluding chains with rmsd greater than 4.5 Å. Dashed line indicate the values obtained for the control set: 28% of modified letter, 0.30 Å rmsd. b) Superimposition of structures with high rmsd and low percentage of modified letters. Chain A of the receptor part of complex 2VIS (hemagglutinin from Influenza virus complexed with immunoglobulin): rmsd = 4.9 Å, 26% of local change. c) Superimposition of structures with low rmsd and many local changes, with corresponding structural letter encoding. Chain A of the receptor part of complex 1I4D (Rac1-GDP complexed with arfaptin from human), rmsd = 0.88 Å, 75% of local change, and chain B of the receptor part of complex 1F51 (transferase complex form *bacillus subtilis*) rmsd = 1.45 Å, 69% of local change. Orange: unbound conformations, green: bound conformations.

To analyze in more details the relation between local and global modifications, we confront the C*α *rmsd with the percentage of structural letter modification in the *complex set*. Figure 2a illustrates the correlation between the number of structural letters that differ between bound and unbound conformations, and the C*α *rmsd between bound and unbound conformations, in the *complex set*. The first is a measure of local structural change, while the second is a measure of global change. It can be seen from Figure 2, that both measures are positively correlated: structures with great rmsd tend to have a high percentage of structural letters that differ between bound and unbound conformations. This confirms the results shown in Table 1. The Pearson correlation coefficient between both measures is 0.59 (p-value is equal to 2.10^-15^). However, protein structures with a C*α *rmsd lower than 1 Å exhibit a great range of structural letter change proportion, from 10 to more than 70%. It indicates that the structural letter sequences bring an information about the structural changes that can not be evaluated by the C*α *rmsd alone.

Two chains exhibit high C*α *rmsd, around 5 Å, but a moderate fraction of structural letter changes, around 30%. These two chains are chain A of receptor part in complex 2VIS (rmsd = 4.9 Å, percentage of change = 26% in a total of 207 structural letters) and chain B of receptor part of the same complex (rmsd = 5.0 Å, percentage of change = 32% in a total of 218 structural letters). The examination of these structures shows that they are made of two domains that undergo large motions upon binding, as can be seen on Figure 2b. The relative orientation of the two domains is significantly modified, hence leading to a high C*α *rmsd. The local structures, however, remains similar, as assessed by the moderate percentage of modified structural letters. If they are superimposed by portion, the two domains have low C*α *rmsd: 0.6 Å and 0.9 Å for domains 1–109 and 110–210 for chain A.

On the contrary, some structures exhibit slight global modifications but a high proportion of local changes: chains A and B of the receptor part of complex 1I4D (respective rmsd are 0.88 and 0.89 Å, respective percentage of modified structural letters are 75 and 68%), and chain B of the receptor part of complex 1F51 (rmsd equal to 1.45 Å, 69% of modified structural letters). These structures, shown on Figure 2c, have a good conservation of there global structures, but the structural letter sequences capture some subtle differences in helix structures. The unbound helices are encoded by runs of helical letter [A], alternate with less regular letters [V] and [W], whereas bound helices are encoded by homogeneous runs of [a], suggesting a higher regularity of bound helices.

It thus appears that the structural alphabet approach offers a complementary approach to the global rmsd as a few local change can be associated to drastic global change, and inversely.

### Comparison with local rmsd

The structural alphabet provides a simplified but detailed description of the protein backbone. As shown on Figure 1 some structural letters have very similar conformation, e.g., [a] and [A], whereas others are clearly different, e.g., [D] and [S]. This disparity has been quantified in [36], by the rmsd_*dev*_: 0.15 Å between [A] and [a], and 1.6 Å between [D] and [S]. Furthermore, the structural letters have different intrinsic variability, as measured by the rmsd_*intra*_. The rmsd_*intra *_of the structural alphabet varies from 0.08 Å for letter [A], to 0.91 Å for letter [F] [36]. The consequences are that (i) different structural encoding can be observed for similar conformations (e. g. a run of [A] replaced by a run of [a]), and (ii) the same structural letter can encode relatively dissimilar fragments, e. g., the most variable letter [F]. It is then desirable to check for consistency between the structural alphabet approach and classical external measures to assess the extend of the local deformations. The aim is to see if the structural alphabet, used for structure description, can also be used to detect significant local deformations.

**Figure 3:**
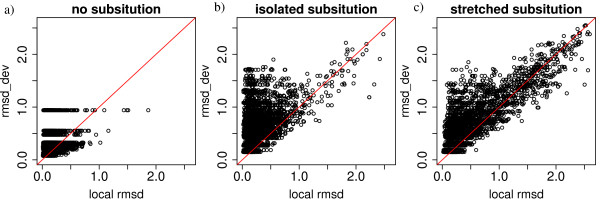
**Comparison of local rmsd and rmsd_*dev*_**. a) Fragments that are encoded by the same structural letter in the bound and unbound forms. The rmsd_*dev *_in this case is the rmsd_*intra*_, which measures the intrinsic variability of structural letters. b) Fragments that are encoded by different structural letters in the bound and unbound forms, in the case where the structural letter substitution is isolated or at the extremity of a stretch of substitutions. c) Fragments that are encoded by different structural letters in the bound and unbound forms, in the case where the structural letter substitution appears in a stretch of substitution. The red line indicates the equality between local rmsd and rmsd_*dev*_.

Structural deformations between bound and unbound forms is usually assessed using classical rmsd computation. In that section, we show a comparison between the assessment of deformation using the structural alphabet and classical rmsd. The measure of deformation using the structural alphabet is given by the rmsd_*dev *_associated to the structural letter change between bound and unbound forms. For the measure using classical rmsd, we computed local C*α *rmsd in a sliding window of four residues along the protein. The reason why we choose a size of four residues for the sliding window is because the structural letters are four-residue long. The results of this comparison on the *complex set *is shown in Figure 3. In case of identical structural letter in the bound and unbound structure (Figure 3a), we consider the rmsd_*intra*_, instead of rmsd_*dev*_, which is a measure of the intrinsic variability of each structural letter. A few cases of identical structural letters correspond to high local rmsd. These cases correspond to fragments that are surrounded by structural letter substitutions. For example, fragment 64–69 of the R chain of the ligand part of complex 1WQ1 is encoded by Q**F**O in the unbound form and F**F**E in the bound form. The fragment encoded by the central F has a local rmsd equal to 1.9Å. The case of different structural letter between bound and unbound forms is called a structural letter substitution (Figure 3b and 3c). Here, we further introduce a distinction between isolated substitutions (Figure 3b) and substitutions that appear in stretch (Figure 3c). An isolated substitution denotes a structural letter that is modified when one or both of its neighbors remain unchanged, e.g, A**R**T → A**B**T or A**R**T → A**B**G. Inversely, a stretched substitution denotes a structural letter change surrounded by modified structural letters, e. g., A**R**T → C**B**G. 61% of the structural letter substitutions appear isolated, and 39% appear in stretch. An unexpected finding of this analysis is that some structural letter substitutions exhibit a high rmsd_*dev *_but a low local rmsd (see Figure 3b and 3c). For example, we observe 534 cases of structural letter substitutions with an associated rmsd_*dev *_greater than 1 Å and a local rmsd lower than 0.5 Å, out of 1,351 substitutions associated to rmsd_*dev *_greater than 1 Å (39%). Among 598 isolated substitutions associated with rmsd _*dev *_greater than 1 Å, 418 correspond to local rmsd lower than 0.5 Å i. e., 70%. If we consider only stretched substitutions, this ratio is only 15% (116 out of 753). This effect is thus more frequently seen in isolated substitutions than in stretched substitutions. This can be globally assessed by the Pearson correlation coefficient between local rmsd and rmsd_*dev*_: 0.50 for isolated substitutions and 0.87 for stretched substitutions. These cases correspond to fragments with low rmsd but encoded by highly dissimilar structural letters. They are due to the stochastic nature of the structural encoding using a HMM.

A consequence is that the rmsd_*dev *_alone cannot be used to quantify the structural change. This analysis tells us that although the structural alphabet offers a unique original tool to detect and qualitatively describe structural deformation, this information has to be combined with the local rmsd in order to properly measure the deformation.

### Analysis of interface regions

The interface regions are defined using Voronoi tessellations. Among the 32,356 residues in the *complex set*, 3,746 are thus defined as interface residues. Interface residues then represent 12% of the whole dataset (i. e. both surface and core residues).

#### Interface local structure

**Figure 4:**
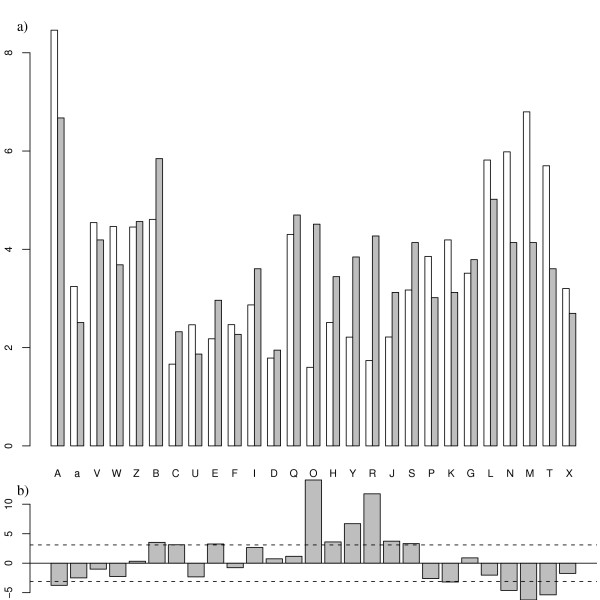
**Local structure composition of the interface set**. a) Percentage of each type of structural letter, in the *complex set *(white) and the *interface set *(gray). b) Z-score to assess the significance of over- or under-representation of the structural letters in the interface. Dashed lines indicate the threshold for statistical significance (-3.1 and 3.1).

The local structure composition of the *interface set *is compared to the global composition of the *complex set *on Figure 4a. The relative frequency of helical letters [A,a,V,W] and extended letters [L,N,M,T,X] is lower in the interface set than in the *complex set*. It is in agreement with the study of Ansari et al [40] who showed that coils are more abundant in protein-protein interfaces than in general. Letters that form helix ends [Z,B,C] are more abundant in the *interface set*. The majority of coil letters are favored at the *interface set *[E,I,D,Q,O,H,Y,R,J,S,G] but some particular letters are not: [U,F,P,K] (strand ends). The statistical significance of structural letter preference for interface regions is assessed by Z-scores computation, illustrated on Figure 4b. These Z-scores compare the structural letter proportion in the interface with the proportion in non-interface regions. The significance threshold, corrected by Bonferroni, is equal to 3.1. Figure 4b indicates that letters [A,K,N,M,T] are significantly under-represented in the interface, while letters [B,C,E,O,H,Y,R,J,S] are significantly over-represented. The structural alphabet thus provides more information than the classical helix/strand/coil classification: some structural letters found in the coil are not favored at the interface.

#### Number of local changes

A total of 3,746 structural letters are involved in the interface: 2,217 (59%) are unchanged and 1,529 (41%) are changed. If we consider the 8 groups of Figure 1, 604 structural letters are changed (16%). This is significantly greater than the results obtained for the whole structures (36% of structural letter changes and 11% with the 8 groups), as assessed by Chi-square tests (p-values < 2.10^-16^).

To assess if each individual letter undergo more substitutions in the *interface set *than in the *control set*, we compute Z-scores (data not shown). All the structural letters, except [a], are more modified in the *interface set *than in the *control set*. The difference is significant for 19 letters out of 27, particularly high for letters I, Q, J, and K.

#### Number of possible substitutions

The number of possible substitutions (Nsub) for each type of structural letters are reported in Table 2. Nsub quantifies, for each structural letter, the mean number of structural letters it can be substituted with. In the *control set*, helical letters [A,a,V,W,Z,B] experience the highest Nsub, around 3–5. It means that these letters are replaced, on average, by three to five different letters. Extended letter [L,N,M,T,X] have Nsub around 2. Among the structural letters that describe coils, only letters [E,F,Y,R,P] have a Nsub greater or equal to 2. It indicates that the expected variations in structural sequences caused by experimental error and natural protein flexibility affect preferentially helices, strands in a lesser extend, and a few coil letters. It is known that loop regions are more flexible that regular secondary structures. The variations observed in helix and strand structural sequences may be due to the fact that our structural alphabet offers a very detailed description of these regions.

**Table 2:** Number of possible substitutions (Nsub) in the different data sets. The numbers between parentheses are the difference between the Nsub and the Nsub of the *control set*.

Structural letter	*Complex set*	*Interface set*	*Control set*
A	4.3 (+ 1.3)	5.1 (+ 2.1)	3.0
a	5.2 (+ 1.1)	4.6 (+ 0.5)	4.1
V	5.7 (+ 1.0)	6.2 (+ 1.5)	4.7
W	5.6 (+ 1.6)	6.3 (+ 2.3)	4.0
Z	5.2 (+ 1.5)	5.9 (+ 2.2)	3.7
B	4.1 (+ 0.6)	4.4 (+ 0.9)	3.5
C	3.4 (+ 1.5)	3.7 (+ 1.8)	1.9
U	2.4 (+ 0.9)	2.3 (+ 0.8)	1.5
E	4.9 (+ 2.6)	5.1 (+ 2.8)	2.3
F	4.7 (+ 1.5)	3.9 (+ 0.7)	3.2
I	2.6 (+ 1.3)	2.9 (+ 1.6)	1.3
D	1.9 (+ 0.7)	2.0 (+ 0.8)	1.2
Q	2.7 (+ 1.1)	3.1 (+ 1.5)	1.6
O	3.3 (+ 1.7)	3.3 (+ 1.7)	1.6
H	2.7 (+ 0.9)	3.8 (+ 2.0)	1.8
Y	3.8 (+ 1.7)	3.5 (+ 1.4)	2.1
R	5.1 (+ 2.5)	5.5 (+ 2.9)	2.6
J	4.8 (+ 3.2)	5.9 (+ 4.3)	1.6
S	2.8 (+ 1.0)	3.4 (+ 1.6)	1.8
P	3.2 (+ 1.0)	3.8 (+ 1.6)	2.2
K	3.1 (+ 1.4)	4.2 (+ 2.5)	1.7
G	2.7 (+ 0.8)	2.8 (+ 0.9)	1.9
L	3.5 (+ 1.2)	4.7 (+ 2.4)	2.3
N	3.5 (+ 1.3)	4.8 (+ 2.6)	2.2
M	2.7 (+ 0.7)	3.2 (+ 1.2)	2.0
T	3.0 (+ 1.0)	4.0 (+ 2.0)	2.0
X	2.8 (+ 1.0)	4.3 (+ 2.5)	1.8

The same global tendency is observed in the *control *and the *interface *sets: high Nsub for helical letters, some of the extended letters and a few coil letters. However, the Nsub computed from the the *complex set *are higher than the Nsub computed from the *control set*. The interface region analysis results, in a majority of cases, in higher Nsub than in the *complex set*, confirming that interface regions undergo more various structural changes. The Nsub are one to two points greater in the *interface set *than in the control set, except for letters [J] (+4.3), [R] (+2.9) and [E] (+2.8), resulting in Nsub greater than 5 for these letters. On the contrary, letter [D] has the lowest Nsub, equal to 2. This analysis thus reveals that some structural letters are particularly affected by the binding (i. e., [E,R,J]).

#### Severity of local deformations

The quantitative measurement of structural letter changes is assessed using the local rmsd. In the control set, 5% of the fragments show a local rmsd greater than 0.2 Å. We will then use 0.2 Å as a threshold to select significant local deformations. In the *complex set*, 25% of the fragments have local rmsd greater than 0.2 Å, and 35% if we restrict to the interface fragments. It thus appears that interface regions undergo more severe local changes than the rest of the structure.

**Figure 5:**
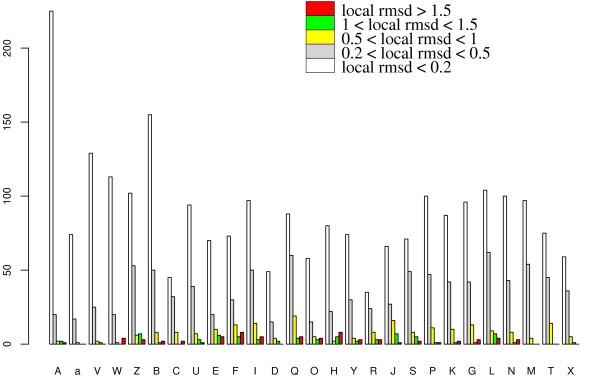
**Severity of local structural change for each structural letter in the interface set**. Histogram of structural letter counts in the unbound conformations, colored according to the severity of the local structural change occurring upon binding, measured by the local rmsd.

Figure 5 presents an analysis of the importance of local deformations in the *interface set *depending on the structural encoding. For each structural letter, we collect the local rmsd and display the number of structural letters affected by a local rmsd in a given range. The unbound form is taken as a reference for this analysis. Helical structural letters [A,a,V,W,Z,B,C] experience very few local rmsd greater than 0.2 Å, indicating that *α*-helices are very stable local structures and are barely affected by the interaction. Among them, letter [Z], which is found at helix borders, shows the highest proportion of local rmsd greater than 0.2 Å. Extended letters [L,N,M,T,X] exhibit higher rmsd, indicating that these local structures are more likely to be modified upon binding, especially letter [L]. The highest number of high local rmsd are observed in coil letters, in particular in letters [E,F,I,Q,J]. Some coil letters, like [U,D,G], are barely affected by the binding.

It thus appears that the severity of local deformation is not uniform among the structural letters, in particular among structural letters describing coils. Some structural letters are more likely to be affected by the formation of protein-protein complex.

#### Structural letter substitutions

**Figure 6:**
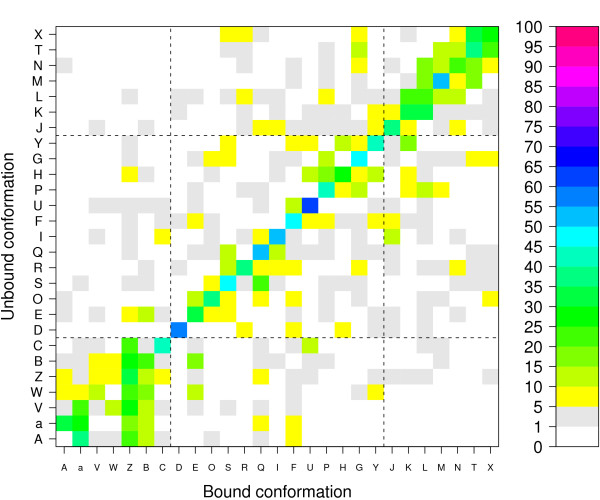
**Probabilities of structural letter substitution associated to local rmsd greater than 0.2Å in the interface set**. The unbound form is taken as the reference for the computation of substitution probabilities.

Now that we have shown that some structural letters are preferentially affected upon binding, the next step is to analyze the resulting conformation after binding, namely the structural letter substitutions. Figure 6 is an illustration of the probabilities of structural letter substitution in the interface region. The unbound form is taken as the reference for this computation. To take into account only significant changes, we restrict the analysis to the pairs of structural letters that correspond to a local rmsd greater than 0.2 Å. The number of structural letter pairs with local rmsd greater than 0.2 Å is 1309, including 488 cases of structural letter identity. Among the 729 possible substitution probabilities (27 × 27), 312 are non-null and 139 are greater than 5%. It must be noted that the substitution probability matrix is highly asymmetrical.

For example, extended letters [A,a,V,W,Z,B,C] display high probabilities to be substituted into letter [Z,B] upon binding. The probability for letter [Z] to be transformed into [V] in the interface region upon binding is 8.8%, whereas it is 28.6% for the inverse transformation from [V] to [Z]. This arises from the normalization with respect to the unbound form needed for the probability computation. The substitution count table is nearly symmetrical, as shown in additional file 1, but the number of structural letters in each class being unequal (see Figure 4), it results in asymmetry in the substitution probabilities. To facilitate the global examination of Figure 6, let us separate the 27 structural letters into the 3 main groups associated to classical secondary structure elements: [a,A,V,W,Z,B,C] for helix and helix borders, [J,K,L,M,N,T,X] for strands and strand borders, and the remaining [D,E,O,S,R,Q,I,F,U,P,H,G,Y] for coils.

Many substitutions occur within the helical group [A,a,V,W,Z,B,C], and, in a lesser extend, in the extended group [L,N,M,T,X]. These substitutions are responsible for subtle modifications of regular secondary structures, like illustrated in Figure 2. Inside the coil group, that gathers a variety of distinct conformations, significant substitutions occur from letters [Y,H,F,R,O] and toward letters [F,Q,S,G]. Eight substitutions with probability greater than 5% are observed from the helix group to the coil group, including 3 substitutions with probabilities greater than 10%: from [C] to [U] (11.9%), from [B] to [E] (18%) and from [W] to [E] (13%). In the same way, 12 substitutions with probability greater than 5% occur from the strand group to the coil group, one of them with probability greater than 10%: from [T] to [G] (10.5%). Four substitutions with probabilities greater than 5% occur from the coil to the helix group (with probability to change from [E] to [B] equal to 15%) and 11 occur from coil to strand group, including three substitutions with high probability: [I] to [J] (11%), [P] to [L] (15%) and [Y] to [K] (15.4%). No substitutions occur between strand and helix groups with high probabilities. This analysis tells us that the local structural modifications that occur upon binding are most of the time subtle substitutions between structural letters belonging to the same major groups. Conformational changes occur mainly following a continuum between helices/coils and strands/coils and a few structural letters play key role in the modifications.

The structural alphabet thus provides a new way to describe local structural changes as the substitution of a structural letter by another one. It is the first time, to our knowledge, that such a qualitative description is reported.

### Graphical examples

**Figure 7:**
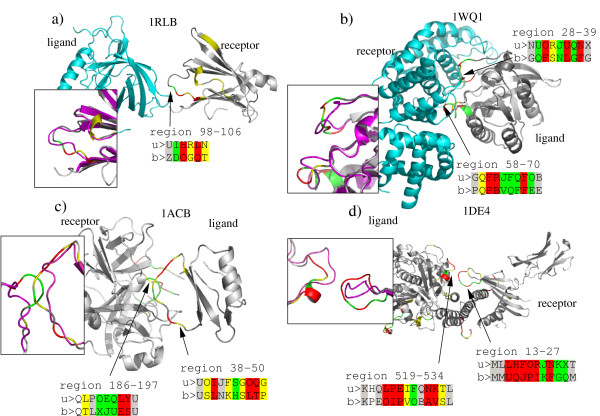
**Examples of local structural changes induced by protein-protein binding**. Proteins are colored according to the rmsd_*dev *_of the letter substitution unbound/bound form, using same color scheme as in Figure 5: white = local rmsd lower than 0.2Å, gray = local rmsd between 0.2 and 0.5 Å, yellow = local rmsd between 0.5 and 1 Å, green = local rmsd between 1 and 1.5 Å, red = local rmsd greater than 1.5 Å. The superimposition of bound and unbound structures (in magenta), is shown for the modified region. The structural encoding are shown for the interface region that are modified: > u: unbound encoding, > b: bound encoding.

Figure 7 illustrates some examples of structural letter substitutions induced by protein-protein interaction. These four complexes are taken from the enzyme-substrate class (Figure 7a, b and 7d) and the other class (Figure 7c). In these four examples, drastic local changes (local rmsd greater than 1.5 Å) occur in the interface regions, within loops. We report the structural sequences of the modified regions, in bound and unbound forms. 1RLB, shown in Figure 7a, is a transthyretin/retinol binding complex. The chain A of transtyretin (the receptor) has a C*α *rmsd equal to 0.7 Å between bound and unbound forms and 47% of the structural letters are modified. A structural modification of region 98–106 includes 2 substitutions associated to local rmsd greater than 1.5 Å: [H] to [O] and [L] to [Q]. A less severe substitution (local rmsd between 1 and 1.5 Å) occurs from [I] and [D]. 1WQ1, shown in Figure 7b, is a ras GTPase/ras GAP complex. Ras GAP (the ligand) is modified in two distinct regions: in loop 28–39 and loop 58–70. Its global C*α *rmsd is 1.1 Å and its percentage of structural letter substitution is 51%. In region 28–39, a run of 7 successive structural letters undergo modifications greater than 0.5 Å, whereas in region 58–70, a run of 8 successive structural letters are strongly modified. Region 58–70 involves letters [J,Q], which are over-modified in interface regions. 1ACB, shown in Figure 7c, is a chymotrypsin/eglin C complex.

Structural modifications occur in both partners: region 186–197 in chymotrypsin and region 38–50 in eglin part. The global C*α *rmsd for chymotrypsin (receptor) is 1.75 Å and the percentage of structural letter substitution is 37%. For eglin (the ligand), global C*α *rmsd is 1.5 Å and 45% of the structural letters are modified. Both modifications involve letters [I,J,Q] which are significantly more modified in the *interface set *than in the *control set*. 1DE4, shown in Figure 7d, is a complex between beta2-microglobulin and a transferrin receptor. The structural modification highlighted here occur in region 13–27 of the beta2-microglobulin and region 519–534 of the transferrin receptor (the ligand). The local structures of both partners are modified were the contact occurs. Beta2-microglobulin (the receptor) has a global C*α *rmsd equal to 1.65 Å and 49% of structural letter substitution. The transferrin receptor (ligand) has a global C*α *rmsd equal to 1.6 Å and 41% of its structural letters are modified upon binding. Both regions involve letters [I,J,K].

**Figure 8:**
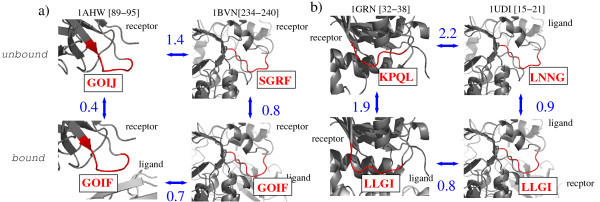
**Examples of local conformational convergence**. Red motifs in rectangles are the structural letter sequences of the fragment highlighted in red in the structures. Numbers associated to blue arrows are the C*α *local rmsd computed between the red fragments. Numbers between brakets denote the region of the protein covered by the structural motif. Top row: unbound structures, bottom: bound structures. a) Motif GOIF is seen in the bound forms of complex 1AHW (antibody-antigene) and 1BVN (enzyme/substrate). b) Motif LLGI is seen in the bound forms of complex 1GRN (other) and 1UDI (enzyme/substrate).

A last illustration of the conformational change analysis using the structural alphabet is shown in Figure 8. In this analysis, we use the structural alphabet to detect common binding motifs in unrelated proteins. The question raised is: " do proteins with unrelated functions exhibit similar structural motifs at the interface ?" The objective is to identity, if any, such structural motifs that could be considered as case of "local structural convergence" toward the same conformation, from unrelated proteins. We applied the following criteria to detect such cases:

• we look for structural motifs at least four structural letter long (i. e., seven residues);

• the motif should be present in the bound forms of at least two complexes from different classes. We consider the 3 classes from Table 3, namely enzyme/substrate, antibody/antigen, and other;

**Table 3:** Description of the complex set

Type (number)	Complexes PDB id
Enzyme-substrate (23)	1ACB, 1AVX, 1AY7, 1BVN, 1CGI, 1D6R, 1DFJ, 1E6E, 1EAW, 1EWY, 1EZU, 1F34, 1HIA, 1KKL, 1MAH, 1PPE, 1TMQ, 1UDI, 2MTA, 2PCC, 2SIC, 2SNI, 7CEI
Antibody-Antigen (10)	1AHW, 1BGX, 1BVK, 1DQJ, 1E6J, 1JPS, 1MLC, 1VFB, 1WEJ, 2VIS
Other (35)	1A2K, 1AK4, 1AKJ, 1ATN, 1B6C, 1BUH, 1DE4, 1E96, 1EER, 1F51, 1FC2, 1FQ1, 1FQJ, 1GCQ, 1GP2, 1GRN, 1H1V, 1HE1, 1HE8, 1I2M, 1I4D, 1IB1, 1IBR, 1IJK, 1KLU, 1KTZ, 1KXP, 1M10, 1ML0, 1N2C, 1QA9, 1RLB, 1SBB, 1WQ1, 2BTF

• the motif should be located in totality at the protein-protein interfaces of the complexes;

• we do not consider runs of helical letters (A,a,V,W,Z,B,C) or extended letters (L,M,N,T,X,J,K). Helices and strands being highly abundant in 3D structures, these motifs may be non significant;

• a significant local deformation should be seen, at the considered motif, in at least one complex;

• the local rmsd between the bound fragments covered by the motif should be lower than the local rmsd between unbound fragments.

Using these criteria, we extracted common bound motifs from proteins with unrelated function. With the rmsd criterion, we select cases where the conformational change induced by the binding put the bound structures closer than the unbound structures, what we call "local structural convergence". Given the limited amount of data we have, and the stringent criteria we applied (in particular, we consider only 3 classes), we found only a few cases of local structural convergence. Two examples are illustrated in Figure 8. Structural motif GOIF is seen in two unrelated complexes: 1AHW, an antibody/antigen complex, and 1BVN, an enzyme/substrate complex. The local C*α *rmsd for the corresponding fragment is 1.4 Å between unbound forms and 0.7 Å only between bound forms. Complex 1AHW undergoes only minor conformational change, as assessed by the rmsd equal to 0.4 Å, and a similar unbound structural motif: GOIJ. Complex 1BVN is modified up to an amount of 0.8 Å, starting from a different structural motif: SGRF. The underlying amino-acid sequences are 'LQHGESP' (1AHW) and 'VIDLGGE' (1BVN). Structural motif LLGI is seen in one 'other' complex, 1GRN (complex between a G-protein and a GTPase activation domain) and one enzyme/substrate complex, 1UDI. Both complexes are significantly modified by the binding: 1.9 Å rmsd for 1GRN, from KPQL to LLGI, and 1UDI, in a lesser extend: 0.9 Å rmsd, from LNNG to LLGI. Local rmsd are equal to 2.2 and 0.8 Å before and after binding respectively. Underlying amino-acid sequences are 'YVPTVFD' (1GRN) and 'QLVIQES' (1UDI). These examples highlight the usefulness of the structural alphabet for further analysis studies using larger data sets.

## Conclusion

This study reveals that the structural alphabet offers a new way to investigate local deformations induced by the protein-protein interaction. Classical studies revealed that interface regions preferentially involve loops. Here, we show that two structural letters forming helix ends [B,C] are preferred at the interface and that only a part of the structural letters describing the loops, [O,H,Y,R,J,S], are preferred at the interface. Letters [E,R,J] are particularly affected by the binding (number of possible substitution greater than 5 *versus *2 in the control set). Concerning the severity of the substitutions, letters [E,F,I,Q,J] are subject to major modifications.

It is the first time that local conformation changes can be qualitatively described in such a way. The main advantage of using the structural alphabet approach, compared to classical rmsd measure, is that it provides a description of bound and unbound conformations, and, in turn, a qualitative description of the deformation. This feature opens the perspective for further studies, such as the classification of interface structural motifs and structural changes. The following questions could be addressed: are the structural modifications common to any type of complexes ? Can the same structural modifications be observed in unrelated proteins ? Could we use the qualitative description of structural changes to make a classification of binding movements ? An example of such analysis is illustrated in Figure 8, in which we highlight two examples of common binding structural motifs from unrelated proteins. Although the actual amount of data is insufficient to derive any conclusive remarks, the structural alphabet approach seems very promising to address such questions.

The computation of structural letter substitution probabilities highlights some preferred substitutions. Such informations could be useful for flexible docking experiments and binding pocket detection at protein surfaces. Flexible docking strategies include the use of ensembles of alternate starting conformations -taken from molecular dynamic simulation [41-44] or other conformational sampling techniques [45]- and the explicit integration of conformational changes during the docking procedure *via *simulated annealing refinement [46] or multicopy mean-field approach [47]. In this framework, the structural letter substitution probabilities derived from the present study could be used in a conformational sampling technique. The structural letter substitution matrix could be used in a generative manner using a Markov process: starting from the unbound structural letter sequence, modifications are introduced using the matrix, to generate a potential bound structural letter sequence. It is then possible to re-build the bound backbone from the structural letter sequence [48,49]. This would probably require some external methods to predict which region is to be modified. The strong transition rules between successive structural letters [36] should also be taken into account in order to generate realistic structural letter sequences.

## Methods

### Dataset

We use the version 2.4 of the benchmark presented by Mintseris et al [50]: 83 crystallographic structures of protein-protein complexes -the bound structures- accompanied by the crystallographic structures of the free ligands and receptors -the unbound structures. The Mintseris dataset consists in 23 enzyme-inhibitor complexes, 21 antibody-antigen complexes (11 of them are in bound/unbound conformation) and 39 other type complexes. As we are interested by structural changes upon binding, the 11 antibody-antigen complexes in bound/unbound conformation are excluded from the analysis. Some ligands and receptors are multichains. The comparison between bound and unbound forms require a correspondence between the residue numbering of each form. This restriction leads to the exclusion of four complexes belonging to the 'other' class. When only the ligand (or the receptor) has inconsistent residue numbering, the receptor (or ligand) is kept in the analysis. Similarly, when one chain of a multimer protein has inconsistent residue numbering, the others were kept in the analysis. 15 chains were then further removed.

The complete dataset of 68 complexes (containing 156 chains from 124 proteins) used in this study is described in Table 3. We will refer to this data set as the *complex set*.

To distinguish the structural deformation induced by protein-protein binding from the experimental uncertainty and the expected variations due to protein flexibility, a *control set *is needed. We consider the control set of 14 protein pairs used by Daily and coworkers [34]:

• 5 protein pairs independently crystallized by different groups: 2CBA/1CAM, 1VDQ/1HEL, 1UNE/1MKT, 1EY0/1STN, and 1TPH/1TPW.

• 9 pairs of allosteric proteins independently crystallized in the same form: 3CHY/1JBE, 1GDD/1BOF, 1GPB/8GPB, 4HHB/1A3N, 1T48/1T49, 1OIW/1YZK, 1VG8/1T91, 1XTS/1XTR, and 2TRT/2TCT.

### Structural alphabet encoding

In this study, the ligand and receptor of each complex, in bound and unbound forms, are simplified into structural letter sequences using HMM-SA and the Viterbi algorithm [35,36]. Local conformational modifications between bound/unbound forms are studied through the structural letter sequences.

### Comparison of bound and unbound structures

**Figure 9:**
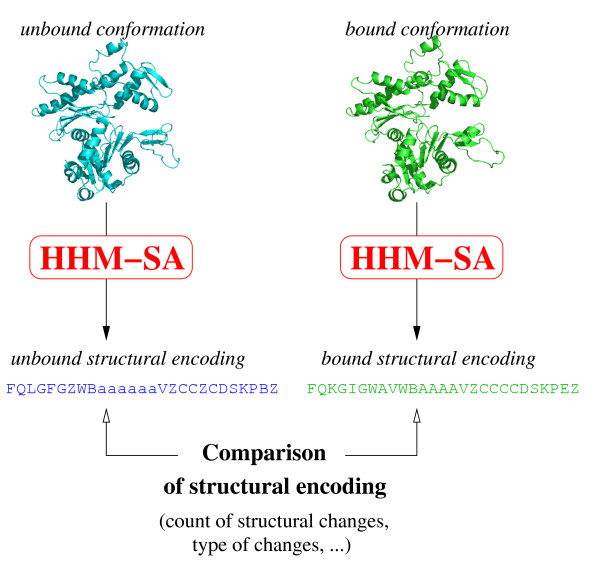
Schematic description of the study.

A total of 156 couples of bound/unbound chains are used to analyze the local structural changes induced by the protein-protein binding. The principle of the study is illustrated in Figure 9.

#### Classical measure of deformation

A classical measure of conformational change is the rmsd (root-mean square deviation), i. e., the mean deviation of atom positions after otimal superimposition of two structures. The rmsd can be computed for the whole protein -a **global rmsd**- or for a fragment of the protein -a **local rmsd**. In this study local and global rmsd are computed using C*α *atoms only, using the ProFit software [51].

#### Deformation assessment by the structural alphabet

As explained in the Results section, a general distance between two different structural letters is given by the **rmsd**_*dev*_, as defined in [36]. The rmsd_*dev *_has been computed from 500 fragment pairs randomly chosen in the two structural letters. The **rmsd**_*intra*_, computed in the same way, measures the intrinsic variability of each structural letter.

The structural distance between two fragments of four residues can then be measured using the local rmsd or the rmsd_*dev*_. Note that the difference between these two rmsd is that the local rmsd is computed for each pair of fragments using proFit, whereas the rmsd_*dev *_is taken from a pre-computed table, by considering only the structural encoding of the fragments.

#### Structural letter substitution probabilities

The use of unbound/bound pairs allows to study the structural modifications as an oriented process: a protein evolves from the unbound state, toward the bound state. The probability to move from letter *x *to letter *y *is then given by:

$$P(x\to y)=\frac{N^{bound}(x\to y)}{N^{unbound}(x)}$$

where *N*^*bound*^(*x *→ *y*) denotes the number of structural letter *x *in the unbound form that are replaced by structural letter *y *in the bound form, and *N*^*unbound*^(*x*) denotes the total number of structural letter *x *in the unbound form. When *x *= *y*, this quantity is the probability of being unchanged. Here, we consider that the unbound state is the starting state and the bound state is the final state. Then, the unbound state will be taken as a reference for the computation, and the resulting matrix might be asymmetrical.

#### Number of possible structural letter substitutions

The number of possible substitutions for each structural letter can then be computed from the substitution probabilities:

*N sub*(*x*) = *e*^*H*(*x*)^

were *H*(*x*) is the Shannon entropy for letter *x*:

$$H(x)=-\sum_{k}P(x\to k)\ln P(x\to k).$$

A *N sub *equal to 1 indicates that structural letter *x *is integrally transformed into one structural letter (it can be itself). The maximum theoretical *N sub *is 27: it means that structural letter *x *is transformed into all the 27 structural letters, with equal probabilities.

### Definition of interface residues

The local modifications induced by protein-protein binding are studied in more details at the receptor-ligand binding interface. Interfaces are detected using Voronoi tessellations. Voronoi tessellations are a way to divide the space around a given set of points into cells. The Voronoi cell around a point contains all the points that are closer to this point than the others. Voronoi tessellations are used to study contacts within proteins, without the use of threshold distance [52]. Here, Voronoi tessellations are used to identify the residues that make contacts between the receptor and the ligand. We use the PROVAT software [53] to compute the Voronoi cells around C*α*, with default parameters. Two residues are in contact if their Voronoi cells share a surface with non-zero area. A structural letter is a four residue fragment. The correspondence is made between a four residue fragment and its third C*α*.

The structural modifications are studied in more details in the interfaces. We will refer to this part of the data as the *interface set*.

### Zscore computation

To study the over-representation of the different structural letters in the interface regions, we compute Zscores defined by:

$$Zscore(x)=\frac{N^{obs}(x)-N^{exp}(x)}{\sqrt{N^{exp}(x)}}.$$

*N*^*obs*^(*x*) denotes the observed number of letter *x *in the *interface set *and *N*^*exp*^(*x*) denotes the expected number of *x *in the interface if the compositions of interface and non-interface regions were similar:

$N^{exp}(x)=f_{\bar{inter}}(x)\times N_{inter}$ were $f_{\bar{inter}}(x)$ denotes the relative frequency of *x *in non-interface region and *N*_*inter *_the number of structural letter of any type in the *interface set*.

Zscores are similarly computed to assess the over-modification of a given structural letter, with *N*^*obs*^(*x*) the number of structural letter *x *that is modified upon binding in the *interface set*, and $N^{exp}(x)=P^{exp}(x\to \bar{x})\times N_{inter}^{unbound}(x)$ where $P^{exp}(x\to \bar{x})$ denotes the probability for letter *x *to be modified in the *control set*, and $N_{inter}^{unbound}(x)$ denotes the number of letter *x *in unbound form in the *interface set*.

Zscores are expected to follow a Gaussian distribution with mean equal to zero and standard deviation of 1. Significance thresholds are corrected to take the multiple tests into account.

## Authors' contributions

JM and HL carried out the comparisons of structural sequences. JM and LR carried out the analysis of the results. ACC and JM conceived the study. All authors read and approved the final manuscript.

## Supplementary Material

Additional file 1 **Structural letter substitution counts in the interface region**. This figure presents the counts of structural letter substitutions corresponding to local rmsd greater than 0.2Å, restricted to the interface region. The normalization with respect to the unbound form results in the substitution probability matrix presented in Figure 6. Click here for file
