# Supplementary material for: Structural deformation upon protein-protein interaction: A structural alphabet approach
Source: BMC Struct Biol. 2008 Feb 28;8:12. doi: 10.1186/1472-6807-8-12 (PMC2315654; doi:10.1186/1472-6807-8-12)
Supplement: Additional file 1 — Structural letter substitution counts in the interface region. This figure presents the counts of structural letter substitutions corresponding to local rmsd greater than 0.2Å, restricted to the interface region. The normalization with respect to the unbound form results in the substitution probability matrix presented in Figure 6. [file 1472-6807-8-12-S1.pdf]

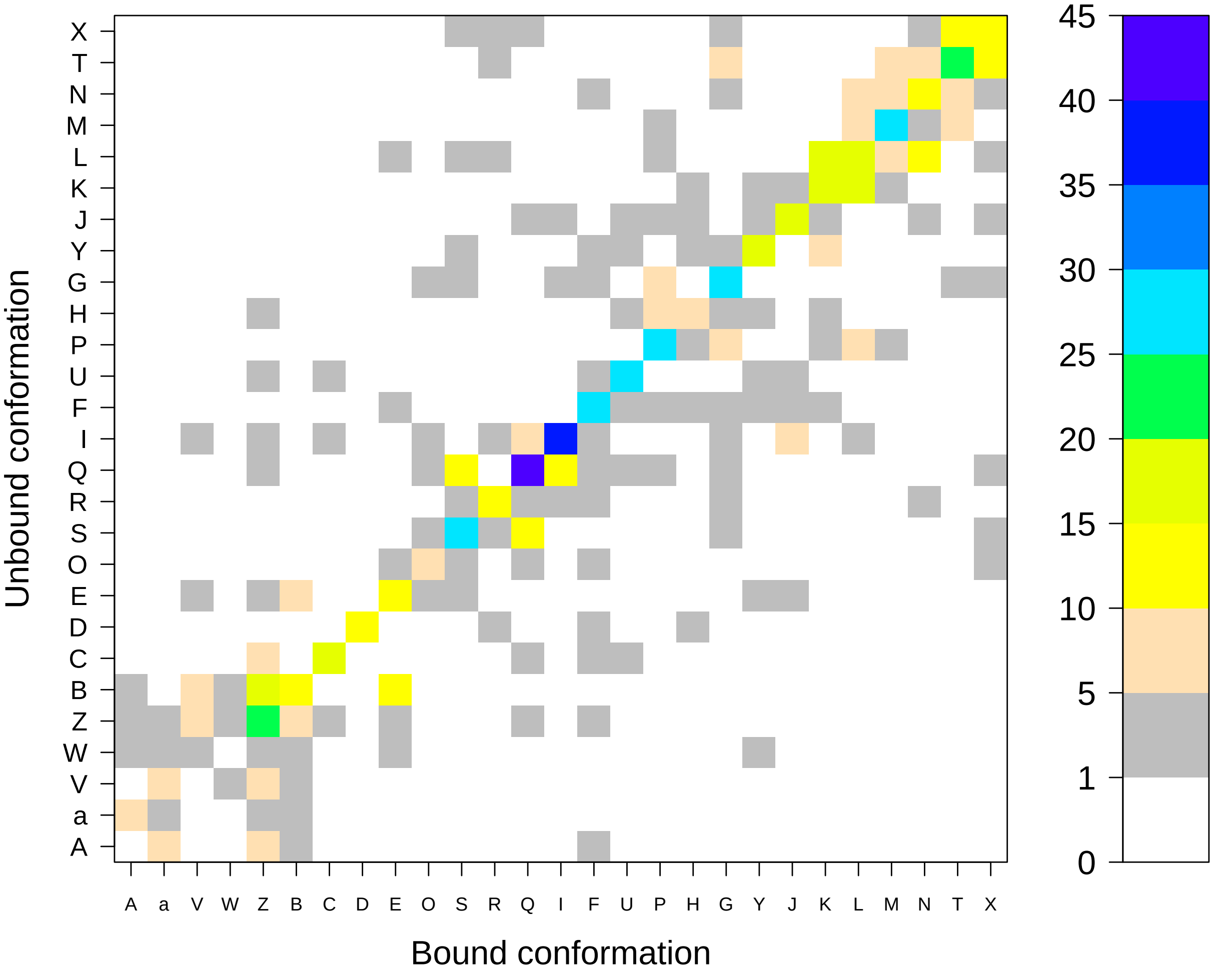

Figure 1: Number of structural letter substitutions corresponding to local rmsd greater than 0.2Å, in the interface region of the complex set. The total number of structural letter pairs is 1309.
